# The Stringent Response-Regulated sRNA Transcriptome of *Borrelia burgdorferi*

**DOI:** 10.3389/fcimb.2018.00231

**Published:** 2018-07-05

**Authors:** Dan Drecktrah, Laura S. Hall, Philipp Rescheneder, Meghan Lybecker, D. Scott Samuels

**Affiliations:** ^1^Division of Biological Sciences, University of Montana, Missoula, MT, United States; ^2^Center for Integrative Bioinformatics Vienna, Max F. Perutz Laboratories, Medical University of Vienna, Vienna, Austria; ^3^Department of Biology, University of Colorado, Colorado Springs, CO, United States; ^4^Center for Biomolecular Structure and Dynamics, University of Montana, Missoula, MT, United States

**Keywords:** Lyme disease, *Borrelia burgdorferi*, stringent response, small RNA, gene regulation, RNA sequencing, guanosine tetraphosphate and pentaphosphate, spirochetes

## Abstract

The Lyme disease spirochete *Borrelia* (*Borreliella*) *burgdorferi* must tolerate nutrient stress to persist in the tick phase of its enzootic life cycle. We previously found that the stringent response mediated by Rel_Bbu_ globally regulates gene expression to facilitate persistence in the tick vector. Here, we show that Rel_Bbu_ regulates the expression of a swath of small RNAs (sRNA), affecting 36% of previously identified sRNAs in *B. burgdorferi*. This is the first sRNA regulatory mechanism identified in any spirochete. Threefold more sRNAs were Rel_Bbu_-upregulated than downregulated during nutrient stress and included antisense, intergenic and 5′ untranslated region sRNAs. Rel_Bbu_-regulated sRNAs associated with genes known to be important for host infection (*bosR* and *dhhp*) as well as persistence in the tick (*glpF* and *hk1*) were identified, suggesting potential mechanisms for post-transcriptional regulation of gene expression.

## Introduction

*Borrelia* (*Borreliella*) *burgdorferi*, the causative agent of Lyme disease (Burgdorfer et al., 1982; Benach et al., 1983; Steere et al., 1983), exists in nature in an enzootic cycle alternating between *Ixodes* ticks and vertebrate hosts (Lane et al., 1991; Radolf et al., 2012; Caimano et al., 2016). Tick larvae acquire *B. burgdorferi* by feeding on an infected host, typically a small mammal (Piesman and Schwan, 2010). *B. burgdorferi* must adapt to persist in the tick midgut during the molt into nymphs and then transmit back to a vertebrate during nymphal feeding (Radolf et al., 2012; Corona and Schwartz, 2015; Caimano et al., 2016). In order to survive these disparate environments, *B. burgdorferi* alters its pattern of gene expression in response to environmental signals, including temperature and nutrients (Radolf et al., 2012; Troxell and Yang, 2013; Iyer and Schwartz, 2016; Samuels and Samuels, 2016; Stevenson and Seshu, 2017). Central to this response is the RpoN-RpoS alternative sigma factor cascade that transcriptionally controls expression of numerous genes required for the transmission from the tick and the establishment of infection in the mammal (Hübner et al., 2001; Fisher et al., 2005; Burtnick et al., 2007; Caimano et al., 2007; Dunham-Ems et al., 2012; Ouyang et al., 2012; Grove et al., 2017). Additionally, the enhancer-binding protein and response regulator Rrp2 (Yang et al., 2003; Burtnick et al., 2007), the transcriptional regulators BosR (Boylan et al., 2003; Hyde et al., 2009; Ouyang et al., 2009, 2011; Katona, 2015) and BadR (Miller et al., 2013; Ouyang and Zhou, 2015), as well as the intracellular second messengers cyclic dimeric GMP (c-di-GMP) (Rogers et al., 2009; Sultan et al., 2010; He et al., 2011; Caimano et al., 2015) and guanosine pentaphosphate and tetraphosphate [(p)ppGpp] (Bugrysheva et al., 2015; Drecktrah et al., 2015) act in concert with RpoN and RpoS to regulate genes required for either host infection or tick persistence (Samuels, 2011; Caimano et al., 2016). However, post-transcriptional regulation of gene expression remains mostly unexplored in *B. burgdorferi* (Lybecker and Samuels, 2007; Jutras et al., 2013; Dulebohn et al., 2014).

Small, noncoding RNAs (sRNAs) have emerged as major regulators of gene expression, although many of the mechanisms are not well understood (Waters and Storz, 2009; Storz et al., 2011; Caldelari et al., 2013; Quereda and Cossart, 2017; Hör et al., 2018). The sRNAs that affect mRNA expression directly are divided into two classes: *cis*-acting, which function in close proximity to their genomic location, and *trans*-acting, which target more distal elements (Georg and Hess, 2011). Both act by binding target mRNAs via complementary base pairing, albeit sometimes with imperfect complementarity (Thomason and Storz, 2010). Antisense RNAs (asRNAs) are a class of sRNAs that are transcribed opposite to the protein-coding sense strand and have complete complementarity to the corresponding sense RNA. Antisense transcripts regulate gene expression through a variety of different mechanisms: transcriptional interference, transcriptional attenuation, translational stimulation or inhibition, and RNA stability, but detailed mechanisms remain elusive despite their ubiquity (Thomason and Storz, 2010; Lybecker et al., 2014). The double-stranded RNA (dsRNA) created by sense-antisense pairing can be cleaved by RNase III (Anacker et al., 2018), which can destabilize or stabilize the RNA, leading to degradation or translation, respectively (Lioliou et al., 2012; Lybecker et al., 2014; Gordon et al., 2017). Thus, levels of asRNA and the associated mRNA may be coordinately upregulated, which suggests the asRNA stabilizes the mRNA, or reciprocally regulated, which suggests destabilization of the mRNA. Riboswitches are another *cis*-acting regulatory RNA. They are usually found in the 5′ untranslated region (5′ UTR) of the mRNA they regulate and function by altering their secondary structure in response to environmental signals and binding intracellular metabolites (Breaker, 2012; Serganov and Nudler, 2013; Sherwood and Henkin, 2016; Nelson and Breaker, 2017). sRNAs transcribed from the intergenic regions between genes (IG RNAs) are typically *trans*-acting sRNAs that have imperfect pairing with their target(s) and regulate the translation and/or degradation of the target RNA (Storz et al., 2011). Thus, a vast transcriptomic armada of noncoding sRNAs exists that provide an additional layer of gene regulation with important implications for post-transcriptional control and, ultimately, protein expression.

The stringent response is invoked by bacteria to adapt and survive nutrient stresses, including decreased levels of amino acids, carbon, phosphate, and iron, by increasing the levels of the intracellular second messenger (p)ppGpp (Chatterji and Ojha, 2001; Potrykus and Cashel, 2008; Liu et al., 2015). This alarmone causes a sea change in physiology, metabolism and gene expression by altering RNA polymerase (RNAP) sigma factor selectivity and associated expression from RpoD-dependent and RpoS-dependent promoters (Dalebroux and Swanson, 2012; Hauryliuk et al., 2015). The enzymes RelA and SpoT, as well as their homologs, control the levels of (p)ppGpp (Potrykus and Cashel, 2008; Atkinson et al., 2011; Dalebroux and Swanson, 2012; Hauryliuk et al., 2015). RelA synthesizes (p)ppGpp and SpoT either synthesizes or hydrolyzes (p)ppGpp in a number of bacteria; however, these two activities are combined in a single bifunctional enzyme, Rel_Bbu_, which mediates the stringent response in *B. burgdorferi* (Concepcion and Nelson, 2003; Bugrysheva et al., 2005; Drecktrah et al., 2015). Rel_Bbu_ globally regulates gene expression to facilitate persistence in the tick during the transition from unfed to fed nymphs when *B. burgdorferi* experiences nutrient deprivation as the blood meal is absorbed in the tick midgut (Drecktrah et al., 2015). Previously, we and others found that expression of the glycerol metabolism (*glp*) operon, which is important for persistence in the tick (He et al., 2011; Pappas et al., 2011), is Rel_Bbu_-dependent (Bugrysheva et al., 2015; Drecktrah et al., 2015). Additionally, the expression of host-associated virulence genes, including *bosR* as well as *dbpBA*, encoding decorin-binding proteins, and *vlsE*, encoding an antigenic variation locus, are regulated by Rel_Bbu_ (Drecktrah et al., 2015).

DsrA, the first regulatory sRNA identified and characterized in *B. burgdorferi*, regulates temperature-dependent translation of the alternative sigma factor RpoS (Lybecker and Samuels, 2007). More recently, small RNA-sequencing analyses of *B. burgdorferi* revealed a vast sRNA landscape containing over 1000 sRNAs, including asRNAs, IG RNAs, and 5′ UTR sRNAs, as well as intraRNAs, which are partial transcripts from the same strand encoding the ORF (Popitsch et al., 2017). Popitsch et al. (2017) described temperature-regulated sRNA expression under conditions that mimic the tick vector (23°C) and mammalian host (37°C) environments, which suggested a complex and dynamic role for sRNAs in the enzootic cycle. Another study identified 351 growth-phase-dependent sRNAs in *B. burgdorferi* (Arnold et al., 2016). Together, these studies have illuminated an impressive tally of sRNAs and have laid the foundation to investigate the regulatory mechanisms for sRNA expression in the Lyme disease spirochete (Lybecker and Samuels, 2017).

In this study, we discovered that the Rel_Bbu_-mediated stringent response is a major regulator of sRNA expression in *B. burgdorferi*, affecting the levels of a third (241 out of 666) of all identified sRNAs (not including intraRNAs). sRNA-regulated gene expression may play a larger regulatory role in this bacterium compared to others as many of the protein-mediated transcriptional regulators and signaling pathways are absent in *B. burgdorferi* (Fraser et al., 1997; Samuels and Radolf, 2009). Herein, we identify sRNAs associated with known virulence factors and metabolic pathways, and we integrate the Rel_Bbu_-dependent sRNA transcriptome and mRNA transcriptome (Drecktrah et al., 2015), evincing implications for sRNA-regulated gene expression in regard to host infectivity and persistence in the vector.

## Materials and methods

### *B. burgdorferi* strains and growth conditions

Low-passage *B. burgdorferi* strain B31-5A4 and *rel*_Bbu_ mutant (Δ*rel*_Bbu_) strains (Drecktrah et al., 2015) were cultured at 35°C in Barbour-Stoenner-Kelly II (BSK) liquid medium, pH 7.6, containing 6% rabbit serum (RS) (Pel-Freez Biologicals) without gelatin (Barbour, 1984; Samuels et al., 2018). For nutrient stress experiments, 40-ml cultures of wild-type and Δ*rel*_Bbu_ *B. burgdorferi* strains were grown as described above until reaching stationary phase (~1 × 10^8^ cells ml^−1^) and collected by centrifugation at 8,000 × g for 10 min at 4°C; the supernatant was discarded and cells were resuspended in RPMI (without glutamate) (Mediatech) for 6 h for the nutrient stress samples. Cell density was determined by enumeration using a Petroff-Hausser cell counting chamber (Samuels et al., 2018).

### RNA isolation

RNA was isolated form *B. burgdorferi* cultures using hot phenol extraction as previously described (Jahn et al., 2008; Drecktrah et al., 2015; Popitsch et al., 2017). RNA was then treated with Turbo DNase according to the manufacturer's instructions. The RNase inhibitor SUPERase-In (Invitrogen) was diluted 1:25 in the reaction mixture and samples were incubated at 37°C for 30 min. DEPC-water was added to bring the total volume up to 300 μl and transferred to a 2.0 ml phase-lock gel heavy tube (5Prime). Three hundred microliter of ultrapure phenol:chloroform:isoamyl was added and samples were inverted 10 times. Samples were centrifuged at 20,800 × g for 7 min at 4°C. Three hundred microliters of chloroform, pH 4.3 (Fisher) was added to the top layer in the phase lock tube and samples inverted 10 times. Samples were centrifuged at 20,800 × g for 7 min at 4°C. The aqueous phase was transferred to a new RNase-free tube and 1 μl of 20 mg ml^−1^ glycogen, 1/10 volume of 3 M sodium acetate, pH 5.2 and, finally, three volumes of ice-cold 100% ethanol were added. Samples were inverted gently to mix and incubated at −20°C overnight. Precipitated RNA was pelleted by centrifugation at 20,800 × g for 45 min at 4°C. The pellet was washed with 75% ethanol and centrifuged at 20,800 × g for 20 min at 4°C. Pellets were dried for 1 min and resuspended in 20 μl of DEPC-water.

### RNA sequencing

RNA sequencing cDNA libraries were generated from two independent biological replicates as previously described (Drecktrah et al., 2015). Importantly, the ribosomal-depleted (Ribo-Zero rRNA removal kit, Illumina) total RNA was fragmented and size-selected (75–300 nucleotides) on an 8% TBE-Urea gel to include both sRNAs and fragmented mRNAs. Libraries were sequenced on an Illumina HiSeq 2000 with single-end 50-base-pair reads at the Vienna Biocenter Core Facilities Next Generation Sequencing unit. As previously described (Popitsch et al., 2017), reads were demultiplexed and adapters were clipped with cutadapt. After quality control, the reads were mapped to the *B. burgdorferi* B31 reference genome with NextGenMap 0.4.10 (Sedlazeck et al., 2013) using standard parameters and a minimum identity threshold of 90%; multireads and reads with a mapping quality smaller than 20 were pruned. FeatureCounts (Liao et al., 2014) was used to calculate raw read counts for all datasets. Annotation of the Rel_Bbu_-dependent sRNAs was based on Popitsch et al. (2017) and gene ontogeny of associated ORFs was guided by the Schutzer annotation (Di et al., 2014). Since the isolated RNA included both small RNAs and fragmented mRNAs, intraRNAs, which are located within annotated genes, could not be distinguished from mRNAs and were not included in the differential sRNA expression analyses. From the read counts, we calculated differential expression between wild type and the *rel*_Bbu_ mutant during nutrient stress using edgeR and filtered the results by adjusted *P*-value ≤ 0.05. To enable inspection in a genome browser, we used CODOC (Popitsch, 2014) to compute strand-specific, normalized depth-of-coverage signals.

### Northern blot analysis

RNA was analyzed by Northern blotting as previously described (Popitsch et al., 2017). Five to fifteen micrograms of RNA was combined with an equal volume of 2 × RNA loading dye and denatured by heating at 65°C for 10 min. Samples were separated on an 8% TBE-Urea gel before transfer to HybondXL membranes (Amersham) by electrotransfer. Membranes were UV-crosslinked and blocked with ULTRAhyb Oligo Hybridization Buffer (Invitrogen) for 1 h at 40°C. Membranes were probed with ^32^P-labeled oligonucleotides overnight at 40°C in a rotating hybridization oven. *ffs*, which encodes the 4.5S RNA of the signal recognition particle, was used as a loading control because 4.5S RNA levels are not dependent on Rel_Bbu_ (Drecktrah et al., 2015). Membranes were washed with 2 × SSC + 0.5% SDS, wrapped in plastic, exposed to an imaging plate for 12–48 h, and analyzed using a Fujifilm FLA-3000G phosphorimager. ^32^P-labeled oligonucleotides (Table S1) were end-labeled with gamma ^32^P-ATP (Perkin-Elmer) and T4 PNK (New England Biolabs) according to the manufacturer's instructions.

## Results and discussion

**Figure 1:**
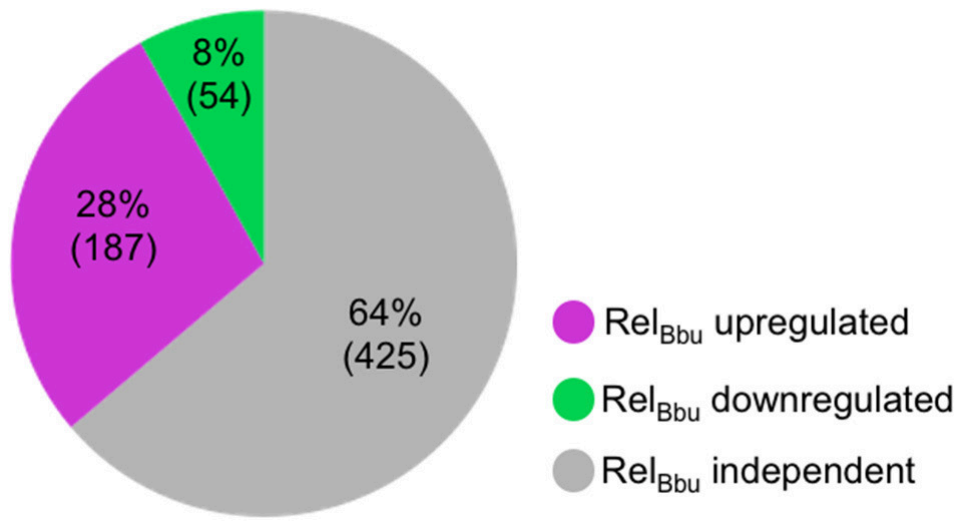
Rel_Bbu_-dependent sRNAs identified by RNA-seq comparing wild-type and Δ*rel*_Bbu_ strains shifted from BSKII + RS to RPMI (nutrient stress). sRNA with greater than twofold upregulation (magenta) or downregulation (green) that were significant (EdgeR p-adjusted of <0.05) were enumerated. The number of sRNAs not affected by Rel_Bbu_ are in gray (Rel_Bbu_ independent). Analyses are based on the annotated sRNAs identified by Popitsch et al. (2017), excluding intraRNAs.

**Figure 2:**
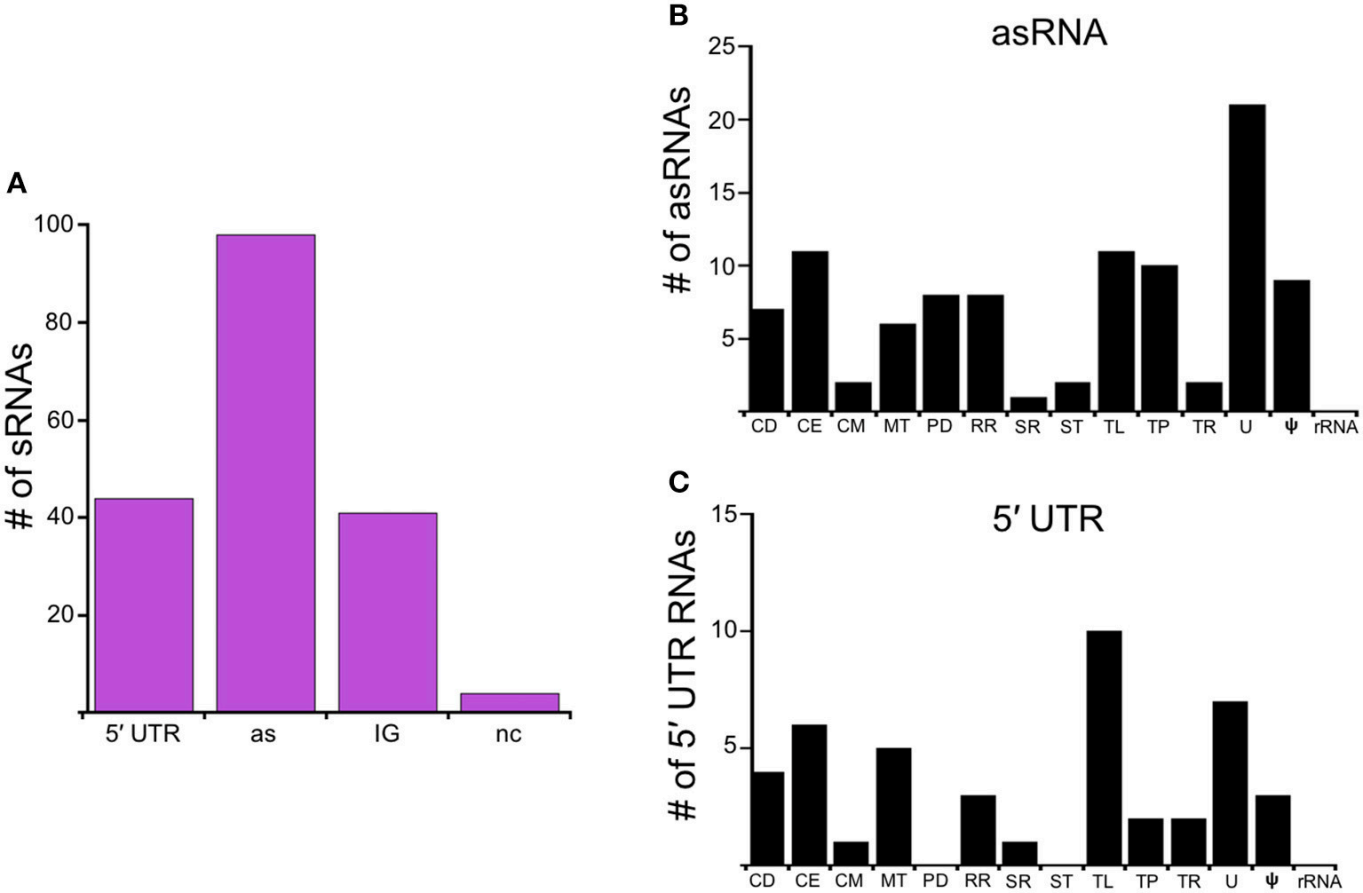
Rel_Bbu_-upregulated sRNAs. **(A)** sRNAs are classified according to their genomic context and include: 5′ untranslated region (5′ UTR), antisense (as), intergenic (IG), and non-coding (nc) RNAs. Functional categories of genes with Rel_Bbu_-upregulated asRNAs **(B)** and 5′ UTR sRNAs **(C)**. Functional categories are abbreviated: cell division (CD), cell envelope (CE), cell motility (CM), metabolism (MT), protein degradation (PD), DNA recombination and repair (RR), stress response (SR), signal transduction (ST), translation (TL), transporter (TP), transcription (TR), unknown (U), pseudogene (ψ), and ribosomal RNA (rRNA).

**Figure 3:**
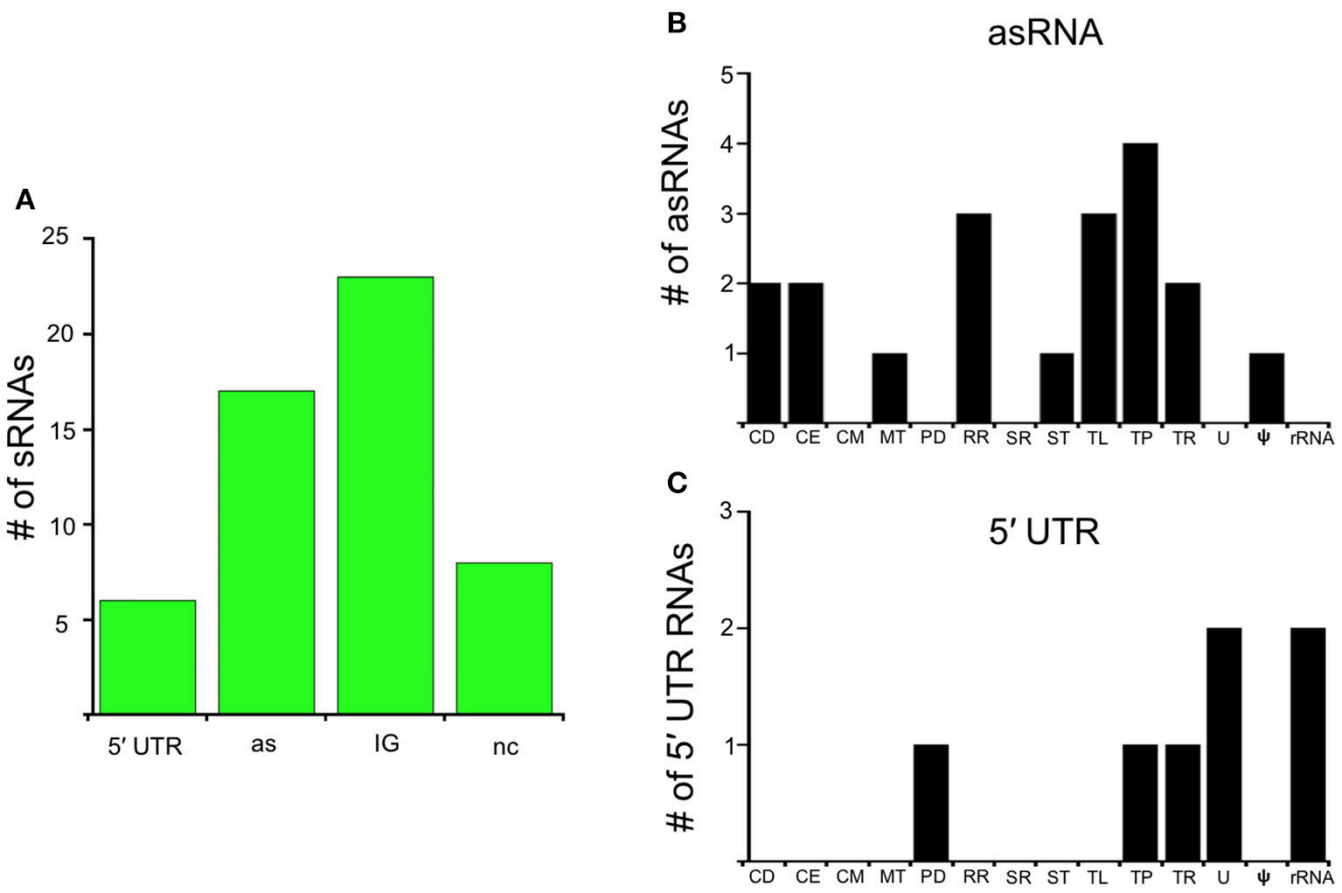
Rel_Bbu_-downregulated sRNAs. **(A)** sRNAs are classified according to their genomic context and include: 5′ untranslated region (5′ UTR), antisense (as), intergenic (IG), and non-coding (nc) RNAs. Functional categories of genes with Rel_Bbu_-downregulated asRNAs **(B)** and 5′ UTR sRNAs **(C)**. Functional categories are abbreviated: cell division (CD), cell envelope (CE), cell motility (CM), metabolism (MT), protein degradation (PD), DNA recombination and repair (RR), stress response (SR), signal transduction (ST), translation (TL), transporter (TP), transcription (TR), unknown (U), pseudogene (ψ), and ribosomal RNA (rRNA).

**Figure 4:**
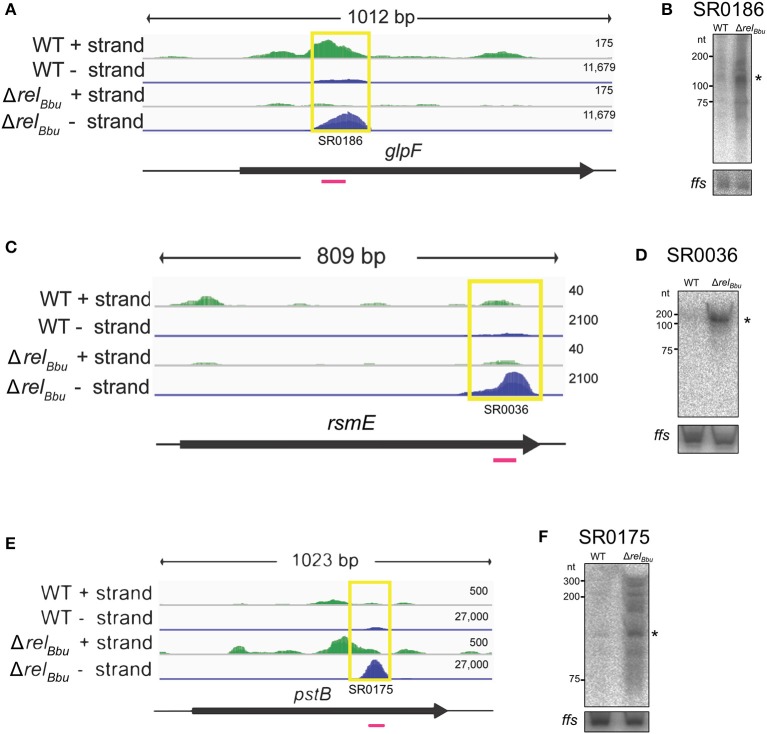
Rel_Bbu_-dependent asRNAs. The deep sequencing reads for *glpF* and *glpF* asRNA (SR0186) **(A)**, *rsmE* and *rsmE* asRNA (SR0036) **(C)**, and *pstB* and *pstB* asRNA (SR0175) **(E)** from both the wild-type and Δ*rel*_Bbu_ strains during nutrient stress are shown in coverage maps with both biological replicates overlaid and displayed in dark and light green (plus strand) and dark and light blue (negative strand). The number associated with each strand represents the normalized number of reads mapped and varies depending on the strand. The corresponding ORFs are shown in black below the RNA-seq coverage maps. The yellow boxes define the position of the called sRNA with the small RNA (SR) number and the magenta lines represent the position of the ^32^P-labeled oligonucleotide probe used in the Northern blot analyses. Northern blot analyses of total RNA from wild-type and Δ*rel*_Bbu_ strains using ^32^P-labeled oligonucleotide probes (Table S1) to the asRNA SR0186 **(B)**, asRNA SR0036 **(D)**, and asRNA SR0175 **(F)**. For each Northern blot, *ffs* (4.5S RNA) was used as a loading control. Asterisks denote the asRNA sizes predicted from RNA-seq analyses.

**Figure 5:**
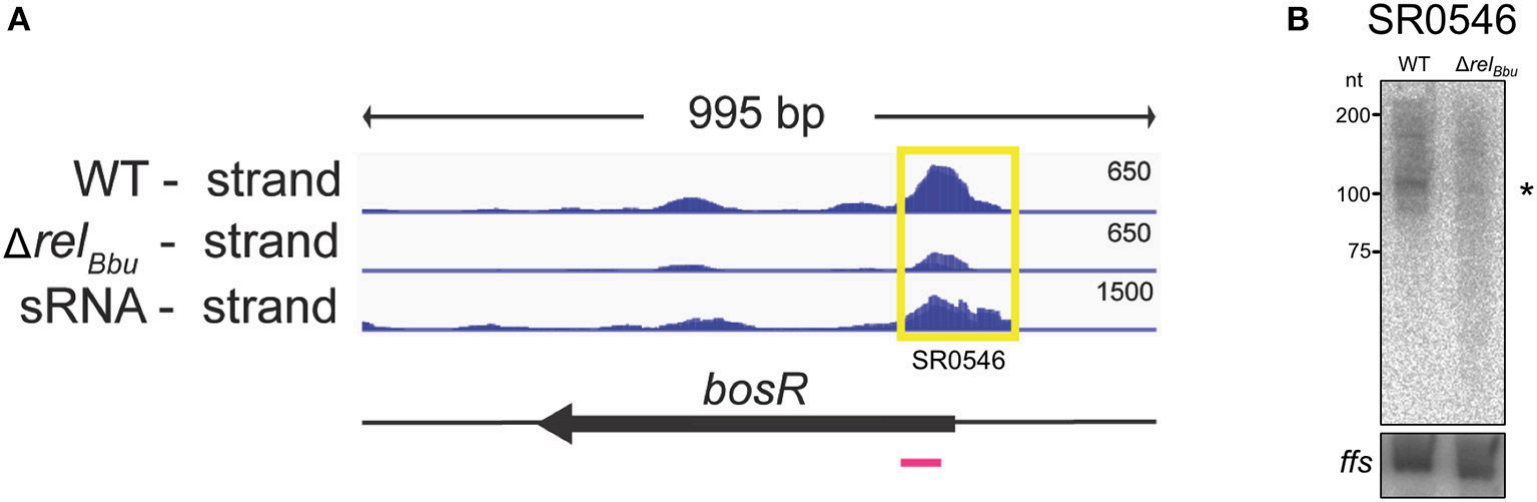
The *bosR* 5′ UTR sRNA (SR0546) is Rel_Bbu_-dependent. **(A)** The deep sequencing reads from both the wild-type and Δ*rel*_Bbu_ strains during nutrient stress are shown in coverage maps with both biological replicates overlaid in dark and light blue (negative strand); the deep sequencing reads from the sRNA coverage map are from Popitsch et al. (2017). The number associated with each strand represents the normalized number of reads and varies depending on the library. The corresponding ORF is shown in black below the RNA-seq coverage maps. The yellow box defines the position of the called sRNA with the SR number and the magenta line represents the position of the ^32^P-labeled oligonucleotide probe used in the Northern blot analysis. **(B)** Northern blot analysis of total RNA from wild-type and Δ*rel*_Bbu_ strains using ^32^P-labeled oligonucleotide probes (Table S1) to the asRNA SR0546 and *ffs* (4.5S RNA) as a loading control. Asterisk denotes the sRNA size predicted from RNA-seq analyses.

In this study, we identify the Rel_Bbu_-dependent sRNA transcriptome under conditions of nutrient stress to better understand gene regulation during persistence of *B. burgdorferi* in its tick vector. We found that Rel_Bbu_ regulates approximately one third of the 666 sRNAs (not including intraRNAs) identified in *B. burgdorferi* by Popitsch et al. (2017), with greater than threefold more sRNAs upregulated than downregulated: 187 sRNAs upregulated (Table S2) and 54 sRNAs downregulated (Table S3) during nutrient stress (Figure 1). sRNAs from each category, IG RNA, asRNA and 5′ UTR sRNA, were Rel_Bbu_-regulated, with asRNAs being the majority of upregulated sRNAs (Figure 2A) and IG RNAs as the largest category of downregulated sRNAs (Figure 3A). Rel_Bbu_-regulated sRNAs were found throughout the genome, including the linear chromosome and the numerous linear and circular plasmids (Fraser et al., 1997; Brisson et al., 2012). sRNAs from all categories with significant and greater than twofold changes in sRNA levels from deep sequencing were validated by Northern blot analyses (Figures 4, 5 and Figures S2–S4). To gain insight into the cellular processes regulated by the Rel_Bbu_ via sRNAs, we categorized the function of the ORFs associated with asRNA and the 5′ UTR sRNAs using biological function gene ontology. Not surprisingly, genes of unknown function were the most common ORF associated with both the upregulated asRNA (Figure 2B) and downregulated 5′ UTR sRNAs (Figure 3C). Interestingly, many asRNAs that were downregulated were opposite genes encoding RNA modification enzymes and other translational machinery (Figure 3B, TL category) and downregulated 5′ UTR sRNAs were associated with rRNA (Figure 3C). Many upregulated 5′ UTR sRNAs were associated with genes encoding translational machinery (Figure 2C, TL category). sRNAs associated with pseudogenes may have important *trans*-acting functions that provide selection for the maintenance of both the sRNA and pseudogene. These results suggest that sRNAs play a previously unrecognized role in the regulation of rRNA expression or stability, which is the original hallmark of the stringent response and the namesake of the *relA* gene (*rela*xed rRNA synthesis). The implications of these initial observations await further investigation.

### *cis*-encoded 5′ UTR sRNAs and asRNAs

5′ UTR sRNAs and asRNAs likely regulate their associated mRNAs, and the mechanism of regulation can be gleaned from comparing their respective Rel_Bbu_-dependent regulation patterns. The regulatory response, either upregulated or downregulated, of Rel_Bbu_ -dependent 5′ UTR sRNAs and asRNAs was compared to the regulatory response of their cognate mRNAs we previously reported (Drecktrah et al., 2015). The number of sRNAs whose cognate mRNA were affected in the same direction, either both upregulated or both downregulated, were designated co-regulated (Figure S1, blue bars), those oppositely affected were termed inversely regulated (Figure S1, orange bars), and the cognate mRNAs that were not Rel_Bbu_-dependent were categorized as not changed (Figure S1, gray bars). Only a single 5′ UTR sRNA was inversely regulated with its cognate mRNA while 19 5′ UTR sRNAs were co-regulated with their associated mRNAs (Figure S1). An equal number of asRNA were co-regulated (15/116) and inversely regulated (15/116) with their associated mRNAs (Figure S1). Most mRNAs associated with the Rel_Bbu_ -regulated 5′ UTR sRNAs and asRNAs were unchanged by Rel_Bbu_ (Figure S1, gray bars); these sRNAs may act in *trans* or may regulate the translation rather than the stability of their mRNA targets. Some 5′ UTR sRNAs may be the termination products of transcriptional riboswitches (Breaker, 2012; Serganov and Nudler, 2013; Sherwood and Henkin, 2016) and, in fact, a ppGpp-sensing riboswitch was recently discovered (Sherlock et al., 2018), suggesting that *B. burgdorferi* may possess a direct RNA-mediated mechanism for regulating gene expression via the stringent response.

### Rel_Bbu_ regulates *glpF* asRNA

Rel_Bbu_ regulates an asRNA associated with the glycerol metabolic (*glp*) operon. Previously, we found that Rel_Bbu_ regulates the *glp* operon, upregulating expression of the glycerol uptake facilitator (*glpF*) and glycerol kinase (*glpK*), while downregulating glycerol 3-phosphate dehydrogenase (*glpD*) (Drecktrah et al., 2015). Our analyses of the Rel_Bbu_-regulated sRNA transcriptome revealed the asRNA SR0186 opposite to *glpF* was downregulated more than threefold by Rel_Bbu_ during nutrient stress (Figure 4A). These results were confirmed by Northern blot analyses (Figure 4B). The *glp* operon encodes gene products responsible for the import and metabolism of glycerol and has been shown to be important for persistence in the tick, where glycerol is thought to be a key carbon source for *B. burgdorferi* (He et al., 2011; Pappas et al., 2011; Corona and Schwartz, 2015; Caimano et al., 2016). Notably, the *glp* operon is a focal point of several *B. burgdorferi* signaling systems, including c-di-GMP, the alternative sigma factor RpoS and the transcriptional activator BosR, which all regulate *glp* transcription in response to environmental signals (Hyde et al., 2006; He et al., 2011; Grove et al., 2017). *glpF* asRNA is a highly expressed sRNA whose levels greatly exceed that of *glpF* mRNA (Figure 4A). The levels of SR0186 asRNA and *glpF* transcript are reciprocally regulated by Rel_Bbu_, suggesting a mechanism in which sense-antisense pairing destabilizes the *glpF* transcript and controls glycerol utilization during the tick phase of the enzootic cycle.

### Rel_Bbu_ regulates sRNAs associated with rRNA methyltransferase genes

rRNAs and tRNAs in the cell are extensively post-transcriptionally modified. Rel_Bbu_ regulates multiple sRNAs associated with genes encoding RNA modifying enzymes. The asRNA SR0036 is opposite *rsmE* (*bb0062*), which encodes a methyltransferase, and the asRNA SR0081 is associated with *bb0129*, which is predicted to encode an rRNA methyltransferase. SR0036 was downregulated by Rel_Bbu_ during nutrient stress (Figures 4C,D), while *rsmE* mRNA was upregulated at stationary phase (Drecktrah et al., 2015), suggesting that the antisense-sense pairing destabilizes the *rsmE* transcript. SR0081 was Rel_Bbu_-upregulated threefold during nutrient stress, although its cognate mRNA (*bb0129*) levels were not significantly changed (Drecktrah et al., 2015). In addition, 5′ UTR sRNAs associated with rRNA methyltransferases were targeted by Rel_Bbu_: the sRNA SR0033 is associated with *bb0052*, which encodes an rRNA/tRNA methyltransferase, and was Rel_Bbu_-upregulated (more than eightfold) during nutrient stress as was the *bb0052* mRNA (more than twofold) (Drecktrah et al., 2015), suggesting that the antisense-sense pairing stabilizes *bb0052* mRNA. The 5′ UTR sRNA SR0226 was Rel_Bbu_-upregulated about fourfold, but there was no significant change in mRNA levels of the associated gene *rrmJ* (*bb0313*), which is predicted to encode a large subunit rRNA methyltransferase (Fraser et al., 1997). Studies in other organisms have revealed a role for rRNA methylation in ribosome biogenesis and ribosome stability/function (Nachtergaele and He, 2017; Sergiev et al., 2018). rRNA methylation in *B. burgdorferi* has not been studied, but our results identifying numerous Rel_Bbu_-regulated sRNAs associated with the genes encoding these modifying enzymes suggest the stringent response controls rRNA modification along with the canonical reduction in rRNA expression.

### Rel_Bbu_ regulates *pstB* asRNA

The stringent response in *B. burgdorferi* also targets two asRNAs linked to genes predicted to encode the high-affinity inorganic phosphate transport (*pst*) operon (Fraser et al., 1997). The first gene, *pstS* (*bb0215*), encodes a substrate-binding protein that was shown to specifically bind inorganic phosphate (Brautigam et al., 2014). The next genes are *pstC* (*bb0216*) and *pstA* (*bb0217*), predicted to encode the permeases, followed by *pstB* (*bb0218*), predicted to encode the nucleotide-binding protein of the phosphate ABC transporter (Fraser et al., 1997). The *pstB* asRNA SR0175 was Rel_Bbu_-downregulated (more than sixfold) under nutrient stress (Figures 4E,F) and *pstB* mRNA followed suit (Drecktrah et al., 2015). Another sRNA, *pstA* asRNA SR0171, was Rel_Bbu_-upregulated while *pstA* transcript was Rel_Bbu_-downregulated (Drecktrah et al., 2015). Thus, our results suggest the *pstB* antisense-sense pairing stabilizes *pstB* mRNA while the *pstA* pairing destabilizes its mRNA. In *E. coli*, expression of the *pst* operon is regulated by the two-component PhoR/PhoB system, as part of the Pho regulon, and is induced in response to inorganic phosphate starvation. The *B. burgdorferi* genome does not contain an annotated PhoR/PhoB two-component regulatory system (Fraser et al., 1997; Galperin, 2010). We propose that the *B. burgdorferi pst* operon, and potentially phosphate import, is regulated by the Rel_Bbu_-dependent sense-antisense pairing, at least under our nutrient stress conditions. At first, downregulation of the phosphate transport system during nutrient stress seems counterintuitive, but our nutrient stress conditions shift *B. burgdorferi* from normal growth medium (BSK + RS) to RPMI, which actually increases inorganic phosphate concentrations more than sixfold. Thus, in our model, nutrient stress conditions, which have increased inorganic phosphate levels, signal Rel_Bbu_-dependent downregulation of *pstB* and *pstA* transcript, potentially via asRNA regulation, presumably resulting in lower PstB and PstA protein levels and less phosphate transport. *pstB* asRNA and *pstB* mRNA are not regulated by temperature (Popitsch et al., 2017), c-di-GMP (Caimano et al., 2015), the transcriptional regulator BadR (Miller et al., 2013), or growth phase (Arnold et al., 2016).

### Rel_Bbu_ regulates the 5′ UTR sRNA of *bosR*

The 5′ UTR sRNA SR0546, which is associated with the gene encoding the transcriptional regulator BosR, was Rel_Bbu_-upregulated (greater than fourfold) during nutrient stress in the RNA-seq data set and validated by Northern blot analyses (Figures 5A,B). BosR is a Fur/PerR homolog that serves as a transcriptional regulator activating the RpoN-RpoS sigma factor cascade that controls the synthesis of a number of host-induced lipoproteins (Boylan et al., 2003; Hyde et al., 2009; Ouyang et al., 2009; Samuels and Radolf, 2009; Katona, 2015). Additionally, BosR has been shown to directly repress expression of genes encoding the tick-phase-specific lipoproteins OspA and OspD during mammalian infection (Wang et al., 2013; Shi et al., 2014). *bosR* expression is regulated not only by Rel_Bbu_ (Drecktrah et al., 2015), but also by the XylR/NagC homolog BadR (Miller et al., 2013; Ouyang and Zhou, 2015) and by its own protein product (Ouyang et al., 2016). The Rel_Bbu_-mediated upregulation of both the *bosR* 5′ UTR sRNA and *bosR* mRNA (each greater than fourfold) during nutrient stress suggests yet another layer of regulation enhancing *bosR* transcription or stability. The mechanistic relationship between the *bosR* 5′ UTR sRNA and mRNA, and how it may affect BosR function, await further investigation.

### Rel_Bbu_ regulates DsrA and 6S RNA

The stringent response also upregulated two annotated sRNAs in *B. burgdorferi*: DsrA (2.6-fold) and 6S RNA (3.3-fold). DsrA, the first regulatory sRNA identified in *B. burgdorferi*, post-transcriptionally regulates the temperature-dependent production of RpoS, the sigma factor that activates genes required for mammalian infection (Lybecker and Samuels, 2007). Interestingly, *rpoS* mRNA levels were upregulated by Rel_Bbu_ in stationary phase, but not during nutrient stress (Drecktrah et al., 2015). Note that DsrA, like other *trans*-encoded sRNAs, may have more than one target RNA.

6S RNA is a widely conserved noncoding RNA that forms a secondary structure mimicking an open promoter complex and binds RNAP, resulting in global transcriptional changes (Wassarman and Storz, 2000; Trotochaud and Wassarman, 2005). 6S RNAs from many bacteria accumulate in stationary growth phase and facilitate the shift from RpoD-dependent to RpoS-dependent promoter gene expression to adapt cells to stationary-phase stresses (Wassarman and Storz, 2000). The predicted *B. burgdorferi* 6S RNA (Bb6S) was identified in a bioinformatic analysis (Barrick et al., 2005) and visualized in RNA-seq studies (Arnold et al., 2016; Popitsch et al., 2017), but its function and regulation were unknown. We found that Bb6S was Rel_Bbu_-upregulated during nutrient stress. This is consistent with the observation that the stringent response increases 6S RNA levels in the soil bacterium *Rhizobium etli* (Vercruysse et al., 2011). Additionally, 6S RNA was found to regulate expression of *relA* and (p)ppGpp levels in *E. coli* (Cavanagh et al., 2010). Thus, our findings augment evidence that the two major transcriptional regulators targeting sigma factor selectivity coordinate their activities to influence global gene expression. The mechanism by which the stringent response alters Bb6S levels remains to be determined, but likely affects transcription, stability or processing/degradation.

### Rel_Bbu_ regulates asRNAs associated with signaling systems

Nucleotide second messengers have central roles in bacterial signaling systems (Pesavento and Hengge, 2009; Kalia et al., 2013; Fahmi et al., 2017; Nelson and Breaker, 2017; Hall and Lee, 2018), and *B. burgdorferi* is no exception (Novak et al., 2014; Caimano et al., 2016; Samuels and Samuels, 2016). Besides for (p)ppGpp and c-di-GMP, cyclic dimeric AMP (c-di-AMP) was recently recognized as an intracellular signal in *B. burgdorferi* (Ye et al., 2014; Savage et al., 2015). DhhP is the essential phosphodiesterase that degrades c-di-AMP, and is required for activation of RpoS (Ye et al., 2014), and CdaA putatively synthesizes c-di-AMP (Savage et al., 2015). SR0008, an asRNA opposite of *cdaA*, is Rel_Bbu_-upregulated sixfold (Table S2) and SR0500, an asRNA opposite of *dhhP*, is Rel_Bbu_-downregulated sixfold (Table S3), which implies a role for the stringent response in regulating c-di-AMP levels via an antisense-sense mechanism in *B. burgdorferi*, although the specific function of c-di-AMP in the spirochete is as of now obscure.

c-di-GMP, which regulates genes required in the tick vector and during enzootic cycle transitions (Novak et al., 2014; Caimano et al., 2016), is synthesized by the diguanlyate cyclase Rrp1(Rogers et al., 2009; He et al., 2011; Sze et al., 2013; Caimano et al., 2015). Rrp1, a two-component response regulator, is activated by its cognate histidine kinase Hk1 (Caimano et al., 2011; Bauer et al., 2015). SR0315 is an asRNA opposite of *hk1* and up-regulated sixfold by Rel_Bbu_ (Table S2). Although the regulatory mechanism has not yet been deciphered, this finding suggests an interaction between the two guanine nucleotide signaling systems as previously hypothesized (Drecktrah et al., 2015).

## Perspectives and conclusions

Only two other (p)ppGpp-dependent (*relA/spoT* or *rel* mutant) bacterial sRNA transcriptomes have been published to our knowledge: *Salmonella enterica* serovar Typhimurium (Ramachandran et al., 2012, 2014; Amin et al., 2016) and the soil bacterium *Rhizobium etli* (Vercruysse et al., 2011). In *Salmonella*, there appear to be more sRNAs downregulated by (p)ppGpp than upregulated, in contrast to *B. burgdorferi* where there are three times more sRNAs upregulated than downregulated by (p)ppGpp. However, these results may depend on the type of environmental conditions inducing the stringent response. Stringent response-regulated sRNAs in *Salmonella* were associated with genes involved in porin expression and resistance to antibiotics (Ramachandran et al., 2014). In *R. etli*, 28 sRNAs were found to be (p)ppGpp-upregulated and five (p)ppGpp-downregulated, with the majority being novel, uncharacterized sRNAs (Vercruysse et al., 2011). Notably, 6S RNA levels were targeted by the stringent response. 6S RNA levels were (p)ppGpp-upregulated in *R. etli* as we found in *B. burgdorferi*, but 6S RNA was downregulated in *Salmonella* (Ramachandran et al., 2014).

The stringent response may not only modulate sRNA levels, but also target RNA-binding proteins, such as the RNA chaperone Hfq, to affect mRNA levels. In *E. coli*, the RelA-mediated stringent response stimulated oligomerization of Hfq monomers to affect the function of sRNAs (Argaman et al., 2012). An unusual homolog of Hfq was identified in *B. burgdorferi* and shown to play a role in host infectivity (Lybecker et al., 2010), but the effect of Rel_Bbu_ on Hfq activity has not yet been examined.

Post-transcriptional sRNA-mediated regulation of mRNA is now established as an important facet of control of gene expression. *B. burgdorferi* has an impressive number of sRNAs that may be paramount in gene regulation to compensate for the dearth of transcriptional activators and repressors as well as two-component systems (Arnold et al., 2016; Adams et al., 2017; Lybecker and Samuels, 2017; Popitsch et al., 2017). Our results demonstrate that the Rel_Bbu_-induced stringent response controls the levels of a third of all known sRNAs (not including intraRNAs). The mechanisms of sRNA regulation and the functional significance of Rel_Bbu_-regulated sRNAs in vertebrate infectivity and persistence in the tick provide an exciting and potentially fruitful avenue for dissecting gene regulation.

## Data availability statement

All sequence files are available from the National Center for Biotechnology Information Sequence Read Archive database (accession numbers SRX971832 and SRX971835).

## Author contributions

DD, ML, and DSS conceived and designed the study. DD, LH, and ML performed the experiments. PR performed the bioinformatics analyses. DD, PR, ML, and DSS interpreted the results. DD wrote the first draft of the manuscript. DD, PR, ML, and DSS wrote sections of the manuscript. All authors read and approved the submitted version.

### Conflict of interest statement

The authors declare that the research was conducted in the absence of any commercial or financial relationships that could be construed as a potential conflict of interest.

## Supplementary material

Figure S1 Correlation of Rel_Bbu_-dependent sRNAs and cognate mRNAs. The mRNAs associated with Rel_Bbu_-dependent 5′ UTR sRNAs or asRNAs were classified as co-regulated (blue bar), inversely regulated (orange bar), or not changed (gray bars) in relation to the sRNA. The correlation was performed using the Rel_Bbu_-regulated mRNA transcriptome from Drecktrah et al. (2015).

Figure S2 The *murJ* asRNA (SR0688) is Rel_Bbu_-dependent. **(A)** The deep sequencing reads from both the wild-type and Δ*rel*_Bbu_ strains during nutrient stress are shown in coverage maps with both biological replicates overlaid in dark and light green (plus strand) and dark and light blue (negative strand). The number associated with each strand represents the normalized number of reads mapped and varies depending on the strand. The corresponding ORFs is shown in black below the RNA-seq mapped reads. The yellow box defines the position of the called sRNA with the SR number and the magenta line represents the position of the ^32^P-labeled oligonucleotide probe used in the Northern blot analyses. **(B)** Northern blot analyses of total RNA from wild-type and Δ*rel*_Bbu_ strains using a ^32^P-labeled oligonucleotide probe (Table S1) to the asRNA SR0688 and *ffs* as a loading control. Asterisk denotes the sRNA size predicted from RNA-seq analyses.

Figure S3 The IG sRNAs SR0725 and SR0735 are Rel_Bbu_-dependent. **(A,C)** The deep sequencing reads from both the wild-type and Δ*rel*_Bbu_ strains during nutrient stress are shown in coverage maps with both biological replicates overlaid in dark and light blue (negative strand). The number associated with each strand represents the normalized number of reads. The corresponding ORFs is shown in black below the RNA-seq coverage maps. The yellow box defines the position of the called sRNA with the SR number and the magenta line represents the position of the ^32^P-labeled oligonucleotide probe used in the Northern blot analyses. **(B,D)** Northern blot analyses of total RNA from wild-type and Δ*rel*_Bbu_ strains using a ^32^P-labeled oligonucleotide probe (Table S1) to the asRNAs SR0725 **(B)** and SR0735 **(D)** and *ffs* as a loading control.

Figure S4 The IG sRNAs SR0961, SR0891, and SR0793 are Rel_Bbu_-dependent. **(A,C,E)** The deep sequencing reads from both the wild-type and Δ*rel*_Bbu_ strains during nutrient stress are shown in coverage maps with both biological replicates overlaid in dark and light green (plus strand). The number associated with each strand represents the normalized number of reads. The corresponding ORFs are shown in black below the RNA-seq coverage maps. The yellow box defines the position of the called sRNA with the SR number and the magenta line represents the position of the ^32^P-labeled oligonucleotide probes used in the Northern blot analyses. **(B,D,F)** Northern blot analyses of total RNA from wild-type and Δ*rel*_Bbu_ strains using a ^32^P-labeled oligonucleotide probe (Table S1) to the asRNAs SR0961 **(B)**, SR0891 **(D)**, and SR0793 **(F)** and *ffs* as a loading control. Asterisks denote the sRNA sizes predicted from RNA-seq analyses.

Table S1 List of sRNAs and oligonucleotides used in Northern blot analyses.

Table S2 List of Rel_Bbu_-upregulated sRNAs.

Table S3 List of Rel_Bbu_-downregulated sRNAs.

## References

[B1] Adams P. P. Flores Avile C. Popitsch N. Bilusic I. Schroeder R. Lybecker M.. (2017). *In vivo* expression technology and 5′ end mapping of the *Borrelia burgdorferi* transcriptome identify novel RNAs expressed during mammalian infection. Nucleic Acids Res. 45, 775–792. 10.1093/nar/gkw1180 27913725 PMC5314773

[B2] Amin S. V. Roberts J. T. Patterson D. G. Coley A. B. Allred J. A. Denner J. M.. (2016). Novel small RNA (sRNA) landscape of the starvation-stress response transcriptome of *Salmonella enterica* serovar typhimurium. RNA Biol. 13, 331–342. 10.1080/15476286.2016.1144010 26853797 PMC4829330

[B3] Anacker M. L. Drecktrah D. LeCoultre R. D. Lybecker M. Samuels D. S. (2018). RNase III processing of rRNA in the Lyme disease spirochete *Borrelia burgdorferi*. J. Bacteriol. 200:e00035-18. 10.1128/JB.00035-18 29632096 PMC5996687

[B4] Argaman L. Elgrably-Weiss M. Hershko T. Vogel J. Altuvia S. (2012). RelA protein stimulates the activity of RyhB small RNA by acting on RNA-binding protein Hfq. Proc. Natl. Acad. Sci. U.S.A. 109, 4621–4626. 10.1073/pnas.1113113109 22393021 PMC3311362

[B5] Arnold W. K. Savage C. R. Brissette C. A. Seshu J. Livny J. Stevenson B. (2016). RNA-seq of *Borrelia burgdorferi* in multiple phases of growth reveals insights into the dynamics of gene expression, transcriptome architecture, and noncoding RNAs. PLoS ONE 11:e0164165. 10.1371/journal.pone.0164165 27706236 PMC5051733

[B6] Atkinson G. C. Tenson T. Hauryliuk V. (2011). The RelA/SpoT homolog (RSH) superfamily: distribution and functional evolution of ppGpp synthetases and hydrolases across the tree of life. PLoS ONE 6:e23479. 10.1371/journal.pone.0023479 21858139 PMC3153485

[B7] Barbour A. G. (1984). Isolation and cultivation of Lyme disease spirochetes. Yale J. Biol. Med. 57, 521–525. 6393604 PMC2589996

[B8] Barrick J. E. Sudarsan N. Weinberg Z. Ruzzo W. L. Breaker R. R. (2005). 6S RNA is a widespread regulator of eubacterial RNA polymerase that resembles an open promoter. RNA 11, 774–784. 10.1261/rna.7286705 15811922 PMC1370762

[B9] Bauer W. J. Luthra A. Zhu G. Radolf J. D. Malkowski M. G. Caimano M. J. (2015). Structural characterization and modeling of the *Borrelia burgdorferi* hybrid histidine kinase Hk1 periplasmic sensor: a system for sensing small molecules associated with tick feeding. J. Struct. Biol. 192, 48–58. 10.1016/j.jsb.2015.08.013 26321039 PMC4605270

[B10] Benach J. L. Bosler E. M. Hanrahan J. P. Coleman J. L. Bast T. F. Habicht G. S.. (1983). Spirochetes isolated from the blood of two patients with Lyme disease. N. Engl. J. Med. 308, 740–742. 10.1056/NEJM198303313081302 6828119

[B11] Boylan J. A. Posey J. E. Gherardini F. C. (2003). *Borrelia* oxidative stress response regulator, BosR: a distinctive Zn-dependent transcriptional activator. Proc. Natl. Acad. Sci. U.S.A. 100, 11684–11689. 10.1073/pnas.2032956100 12975527 PMC208818

[B12] Brautigam C. A. Ouyang Z. Deka R. K. Norgard M. V. (2014). Sequence, biophysical, and structural analyses of the PstS lipoprotein (BB0215) from *Borrelia burgdorferi* reveal a likely binding component of an ABC-type phosphate transporter. Protein Sci. 23, 200–212. 10.1002/pro.2406 24318969 PMC3926745

[B13] Breaker R. R. (2012). Riboswitches and the RNA world. Cold Spring Harb. Perspect. Biol. 4:a003566. 10.1101/cshperspect.a003566 21106649 PMC3281570

[B14] Brisson D. Drecktrah D. Eggers C. H. Samuels D. S. (2012). Genetics of *Borrelia burgdorferi*. Annu. Rev. Genet. 46, 515–536. 10.1146/annurev-genet-011112-112140 22974303 PMC3856702

[B15] Bugrysheva J. V. Bryksin A. V. Godfrey H. P. Cabello F. C. (2005). *Borrelia burgdorferi rel* is responsible for generation of guanosine-3′-diphosphate-5′-triphosphate and growth control. Infect. Immun. 73, 4972–4981. 10.1128/IAI.73.8.4972-4981.2005 16041012 PMC1201186

[B16] Bugrysheva J. V. Pappas C. J. Terekhova D. A. Iyer R. Godfrey H. P. Schwartz I.. (2015). Characterization of the Rel_Bbu_ regulon in *Borrelia burgdorferi* reveals modulation of glycerol metabolism by (p)ppGpp. PLoS ONE 10:e0118063. 10.1371/journal.pone.0118063 25688856 PMC4331090

[B17] Burgdorfer W. Barbour A. G. Hayes S. F. Benach J. L. Grunwaldt E. Davis J. P. (1982). Lyme disease–a tick-borne spirochetosis? Science 216, 1317–1319. 7043737 10.1126/science.7043737

[B18] Burtnick M. N. Downey J. S. Brett P. J. Boylan J. A. Frye J. G. Hoover T. R.. (2007). Insights into the complex regulation of *rpoS* in *Borrelia burgdorferi*. Mol. Microbiol. 65, 277–293. 10.1111/j.1365-2958.2007.05813.x 17590233 PMC1976401

[B19] Caimano M. J. Drecktrah D. Kung F. Samuels D. S. (2016). Interaction of the Lyme disease spirochete with its tick vector. Cell. Microbiol. 18, 919–927. 10.1111/cmi.12609 27147446 PMC5067140

[B20] Caimano M. J. Dunham-Ems S. Allard A. M. Cassera M. B. Kenedy M. Radolf J. D. (2015). Cyclic di-GMP modulates gene expression in Lyme disease spirochetes at the tick-mammal interface to promote spirochete survival during the blood meal and tick-to-mammal transmission. Infect. Immun. 83, 3043–3060. 10.1128/IAI.00315-15 25987708 PMC4496621

[B21] Caimano M. J. Iyer R. Eggers C. H. Gonzalez C. Morton E. A. Gilbert M. A.. (2007). Analysis of the RpoS regulon in *Borrelia burgdorferi* in response to mammalian host signals provides insight into RpoS function during the enzootic cycle. Mol. Microbiol. 65, 1193–1217. 10.1111/j.1365-2958.2007.05860.x 17645733 PMC2967192

[B22] Caimano M. J. Kenedy M. R. Kairu T. Desrosiers D. C. Harman M. Dunham-Ems S.. (2011). The hybrid histidine kinase Hk1 is part of a two-component system that is essential for survival of *Borrelia burgdorferi* in feeding *Ixodes scapularis* ticks. Infect. Immun. 79, 3117–3130. 10.1128/IAI.05136-11 21606185 PMC3147546

[B23] Caldelari I. Chao Y. Romby P. Vogel J. (2013). RNA-mediated regulation in pathogenic bacteria. Cold Spring Harb. Perspect. Med. 3:a010298. 10.1101/cshperspect.a010298 24003243 PMC3753719

[B24] Cavanagh A. T. Chandrangsu P. Wassarman K. M. (2010). 6S RNA regulation of *relA* alters ppGpp levels in early stationary phase. Microbiology 156, 3791–3800. 10.1099/mic.0.043992-0 20829285 PMC3068707

[B25] Chatterji D. Ojha A. K. (2001). Revisiting the stringent response, ppGpp and starvation signaling. Curr. Opin. Microbiol. 4, 160–165. 10.1016/S1369-5274(00)00182-X 11282471

[B26] Concepcion M. B. Nelson D. R. (2003). Expression of *spoT* in *Borrelia burgdorferi* during serum starvation. J. Bacteriol. 185, 444–452. 10.1128/JB.185.2.444-452.2003 12511489 PMC145309

[B27] Corona A. Schwartz I. (2015). *Borrelia burgdorferi*: carbon metabolism and the tick-mammal enzootic cycle. Microbiol. Spectr. 3:MBP-0011-2014. 10.1128/microbiolspec.MBP-0011-2014 26185064 PMC7942402

[B28] Dalebroux Z. D. Swanson M. S. (2012). ppGpp: magic beyond RNA polymerase. Nat. Rev. Microbiol. 10, 203–212. 10.1038/nrmicro2720 22337166 PMC13198741

[B29] Di L. Pagan P. E. Packer D. Martin C. L. Akther S. Ramrattan G.. (2014). *BorreliaBase*: a phylogeny-centered browser of *Borrelia* genomes. BMC Bioinformatics 15:233. 10.1186/1471-2105-15-233 24994456 PMC4094996

[B30] Drecktrah D. Lybecker M. Popitsch N. Rescheneder P. Hall L. S. Samuels D. S. (2015). The *Borrelia burgdorferi* RelA/SpoT homolog and stringent response regulate survival in the tick vector and global gene expression during starvation. PLoS Pathog. 11:e1005160. 10.1371/journal.ppat.1005160 26371761 PMC4570706

[B31] Dulebohn D. P. Hayes B. M. Rosa P. A. (2014). Global repression of host-associated genes of the Lyme disease spirochete through post-transcriptional modulation of the alternative sigma factor RpoS. PLoS ONE 9:e93141. 10.1371/journal.pone.0093141 24671196 PMC3966842

[B32] Dunham-Ems S. M. Caimano M. J. Eggers C. H. Radolf J. D. (2012). *Borrelia burgdorferi* requires the alternative sigma factor RpoS for dissemination within the vector during tick-to-mammal transmission. PLoS Pathog. 8:e1002532. 10.1371/journal.ppat.1002532 22359504 PMC3280991

[B33] Fahmi T. Port G. C. Cho K. H. (2017). c-di-AMP: an essential molecule in the signaling pathways that regulate the viability and virulence of gram-positive bacteria. Genes 8:197. 10.3390/genes8080197 28783096 PMC5575661

[B34] Fisher M. A. Grimm D. Henion A. K. Elias A. F. Stewart P. E. Rosa P. A.. (2005). *Borrelia burgdorferi* σ^54^ is required for mammalian infection and vector transmission but not for tick colonization. Proc. Natl. Acad. Sci. U.S.A. 102, 5162–5167. 10.1073/pnas.0408536102 15743918 PMC555983

[B35] Fraser C. M. Casjens S. Huang W. M. Sutton G. G. Clayton R. Lathigra R.. (1997). Genomic sequence of a Lyme disease spirochete, *Borrelia burgdorferi*. Nature 390, 580–586. 10.1038/37551 9403685

[B36] Galperin M. Y. (2010). Diversity of structure and function of response regulator output domains. Curr. Opin. Microbiol. 13, 150–159. 10.1016/j.mib.2010.01.005 20226724 PMC3086695

[B37] Georg J. Hess W. R. (2011). *cis*-antisense RNA, another level of gene regulation in bacteria. Microbiol. Mol. Biol. Rev. 75, 286–300. 10.1128/MMBR.00032-10 21646430 PMC3122628

[B38] Gordon G. C. Cameron J. C. Pfleger B. F. (2017). RNA sequencing identifies new RNase III cleavage sites in *Escherichia coli* and reveals increased regulation of mRNA. MBio 8:e00128-17. 10.1128/mBio.00128-17 28351917 PMC5371410

[B39] Grove A. P. Liveris D. Iyer R. Petzke M. Rudman J. Caimano M. J.. (2017). Two distinct mechanisms govern RpoS-mediated repression of tick-phase genes during mammalian host adaptation by *Borrelia burgdorferi*, the Lyme disease spirochete. MBio 8:e01204-17. 10.1128/mBio.01204-17 28830947 PMC5565969

[B40] Hall C. L. Lee V. T. (2018). Cyclic-di-GMP regulation of virulence in bacterial pathogens. Wiley Interdiscip. Rev. RNA 9:e1454. 10.1002/wrna.1454 28990312 PMC5739959

[B41] Hauryliuk V. Atkinson G. C. Murakami K. S. Tenson T. Gerdes K. (2015). Recent functional insights into the role of (p)ppGpp in bacterial physiology. Nat. Rev. Microbiol. 13, 298–309. 10.1038/nrmicro3448 25853779 PMC4659695

[B42] He M. Ouyang Z. Troxell B. Xu H. Moh A. Piesman J.. (2011). Cyclic di-GMP is essential for the survival of the Lyme disease spirochete in ticks. PLoS Pathog. 7:e1002133. 10.1371/journal.ppat.1002133 21738477 PMC3128128

[B43] Hör J. Gorski S. A. Vogel J. (2018). Bacterial RNA biology on a genome scale. Mol. Cell 70, 785–799. 10.1016/j.molcel.2017.12.023 29358079

[B44] Hübner A. Yang X. Nolen D. M. Popova T. G. Cabello F. C. Norgard M. V. (2001). Expression of *Borrelia burgdorferi* OspC and DbpA is controlled by a RpoN-RpoS regulatory pathway. Proc. Natl. Acad. Sci. U.S.A. 98, 12724–12729. 10.1073/pnas.231442498 11675503 PMC60121

[B45] Hyde J. A. Seshu J. Skare J. T. (2006). Transcriptional profiling of *Borrelia burgdorferi* containing a unique *bosR* allele identifies a putative oxidative stress regulon. Microbiology 152, 2599–2609. 10.1099/mic.0.28996-0 16946255

[B46] Hyde J. A. Shaw D. K. Smith R. III. Trzeciakowski J. P. Skare J. T. (2009). The BosR regulatory protein of *Borrelia burgdorferi* interfaces with the RpoS regulatory pathway and modulates both homeostatic and pathogenic properties of the Lyme disease spirochete. Mol. Microbiol. 74, 1344–1355. 10.1111/j.1365-2958.2009.06951.x 19906179 PMC2805275

[B47] Iyer R. Schwartz I. (2016). Microarray-based comparative genomic and transcriptome analysis of *Borrelia burgdorferi*. Microarrays 5:9. 10.3390/microarrays5020009 27600075 PMC5003485

[B48] Jahn C. E. Charkowski A. O. Willis D. K. (2008). Evaluation of isolation methods and RNA integrity for bacterial RNA quantitation. J. Microbiol. Methods 75, 318–324. 10.1016/j.mimet.2008.07.004 18674572

[B49] Jutras B. L. Jones G. S. Verma A. Brown N. A. Antonicello A. D. Chenail A. M.. (2013). Posttranscriptional self-regulation by the Lyme disease bacterium's BpuR DNA/RNA-binding protein. J. Bacteriol. 195, 4915–4923. 10.1128/JB.00819-13 23974034 PMC3807498

[B50] Kalia D. Merey G. Nakayama S. Zheng Y. Zhou J. Luo Y.. (2013). Nucleotide, c-di-GMP, c-di-AMP, cGMP, cAMP, (p)ppGpp signaling in bacteria and implications in pathogenesis. Chem. Soc. Rev. 42, 305–341. 10.1039/C2CS35206K 23023210

[B51] Katona L. I. (2015). The Fur homologue BosR requires Arg39 to activate *rpoS* transcription in *Borrelia burgdorferi* and thereby direct spirochaete infection in mice. Microbiology 161, 2243–2255. 10.1099/mic.0.000166 26318670 PMC4806591

[B52] Lane R. S. Piesman J. Burgdorfer W. (1991). Lyme borreliosis: relation of its causative agent to its vectors and hosts in North America and Europe. Annu. Rev. Entomol. 36, 587–609. 10.1146/annurev.en.36.010191.003103 2006870

[B53] Liao Y. Smyth G. K. Shi W. (2014). featureCounts: an efficient general purpose program for assigning sequence reads to genomic features. Bioinformatics 30, 923–930. 10.1093/bioinformatics/btt656 24227677

[B54] Lioliou E. Sharma C. M. Caldelari I. Helfer A. C. Fechter P. Vandenesch F.. (2012). Global regulatory functions of the *Staphylococcus aureus* endoribonuclease III in gene expression. PLoS Genet. 8:e1002782. 10.1371/journal.pgen.1002782 22761586 PMC3386247

[B55] Liu K. Bittner A. N. Wang J. D. (2015). Diversity in (p)ppGpp metabolism and effectors. Curr. Opin. Microbiol. 24, 72–79. 10.1016/j.mib.2015.01.012 25636134 PMC4380541

[B56] Lybecker M. C. Abel C. A. Feig A. L. Samuels D. S. (2010). Identification and function of the RNA chaperone Hfq in the Lyme disease spirochete *Borrelia burgdorferi*. Mol. Microbiol. 78, 622–635. 10.1111/j.1365-2958.2010.07374.x 20815822 PMC2963666

[B57] Lybecker M. C. Samuels D. S. (2007). Temperature-induced regulation of RpoS by a small RNA in *Borrelia burgdorferi*. Mol. Microbiol. 64, 1075–1089. 10.1111/j.1365-2958.2007.05716.x 17501929

[B58] Lybecker M. C. Samuels D. S. (2017). Small RNAs of *Borrelia burgdorferi*: characterizing functional regulators in a sea of sRNAs. Yale J. Biol. Med. 90, 317–323. 28656017 PMC5482307

[B59] Lybecker M. Bilusic I. Raghavan R. (2014). Pervasive transcription: detecting functional RNAs in bacteria. Transcription 5:e944039. 10.4161/21541272.2014.944039 25483405 PMC4581347

[B60] Miller C. L. Karna S. L. Seshu J. (2013). *Borrelia* host adaptation Regulator (BadR) regulates *rpoS* to modulate host adaptation and virulence factors in *Borrelia burgdorferi*. Mol. Microbiol. 88, 105–124. 10.1111/mmi.12171 23387366 PMC4828661

[B61] Nachtergaele S. He C. (2017). The emerging biology of RNA post-transcriptional modifications. RNA Biol. 14, 156–163. 10.1080/15476286.2016.1267096 27937535 PMC5324755

[B62] Nelson J. W. Breaker R. R. (2017). The lost language of the RNA world. Sci. Signal. 10:eaam8812. 10.1126/scisignal.aam8812 28611182 PMC5789781

[B63] Novak E. A. Sultan S. Z. Motaleb M. A. (2014). The cyclic-di-GMP signaling pathway in the Lyme disease spirochete, *Borrelia burgdorferi*. Front. Cell. Infect. Microbiol. 4:56. 10.3389/fcimb.2014.00056 24822172 PMC4013479

[B64] Ouyang Z. Zhou J. (2015). BadR (BB0693) controls growth phase-dependent induction of *rpoS* and *bosR* in *Borrelia burgdorferi* via recognizing TAAAATAT motifs. Mol. Microbiol. 98, 1147–1167. 10.1111/mmi.13206 26331438

[B65] Ouyang Z. Deka R. K. Norgard M. V. (2011). BosR (BB0647) controls the RpoN-RpoS regulatory pathway and virulence expression in *Borrelia burgdorferi* by a novel DNA-binding mechanism. PLoS Pathog. 7:e1001272. 10.1371/journal.ppat.1001272 21347346 PMC3037356

[B66] Ouyang Z. Kumar M. Kariu T. Haq S. Goldberg M. Pal U.. (2009). BosR (BB0647) governs virulence expression in *Borrelia burgdorferi*. Mol. Microbiol. 74, 1331–1343. 10.1111/j.1365-2958.2009.06945.x 19889086 PMC2831293

[B67] Ouyang Z. Narasimhan S. Neelakanta G. Kumar M. Pal U. Fikrig E.. (2012). Activation of the RpoN-RpoS regulatory pathway during the enzootic life cycle of *Borrelia burgdorferi*. BMC Microbiol. 12:44. 10.1186/1471-2180-12-44 22443136 PMC3320556

[B68] Ouyang Z. Zhou J. Norgard M. V. (2016). Evidence that BosR (BB0647) Is a positive autoregulator in *Borrelia burgdorferi*. Infect. Immun. 84, 2566–2574. 10.1128/IAI.00297-16 27324485 PMC4995921

[B69] Pappas C. J. Iyer R. Petzke M. M. Caimano M. J. Radolf J. D. Schwartz I. (2011). *Borrelia burgdorferi* requires glycerol for maximum fitness during the tick phase of the enzootic cycle. PLoS Pathog. 7:e1002102. 10.1371/journal.ppat.1002102 21750672 PMC3131272

[B70] Pesavento C. Hengge R. (2009). Bacterial nucleotide-based second messengers. Curr. Opin. Microbiol. 12, 170–176. 10.1016/j.mib.2009.01.007 19318291

[B71] Piesman J. Schwan T. G. (2010). Ecology of borreliae and their arthropod vectors, in Borrelia: Molecular Biology, Host Interaction and Pathogenesis, eds Samuels D. S. Radolf J. D. (Norfolk: Caister Academic Press), 251–278.

[B72] Popitsch N. (2014). CODOC: efficient access, analysis and compression of depth of coverage signals. Bioinformatics 30, 2676–2677. 10.1093/bioinformatics/btu362 24872424

[B73] Popitsch N. Bilusic I. Rescheneder P. Schroeder R. Lybecker M. (2017). Temperature-dependent sRNA transcriptome of the Lyme disease spirochete. BMC Genomics 18:28. 10.1186/s12864-016-3398-3 28056764 PMC5216591

[B74] Potrykus K. Cashel M. (2008). (p)ppGpp: still magical? Annu. Rev. Microbiol. 62, 35–51. 10.1146/annurev.micro.62.081307.162903 18454629

[B75] Quereda J. J. Cossart P. (2017). Regulating bacterial virulence with RNA. Annu. Rev. Microbiol. 71, 263–280. 10.1146/annurev-micro-030117-020335 28886688

[B76] Radolf J. D. Caimano M. J. Stevenson B. Hu L. T. (2012). Of ticks, mice and men: understanding the dual-host lifestyle of Lyme disease spirochaetes. Nat. Rev. Microbiol. 10, 87–99. 10.1038/nrmicro2714 22230951 PMC3313462

[B77] Ramachandran V. K. Shearer N. Thompson A. (2014). The primary transcriptome of *Salmonella enterica* serovar Typhimurium and its dependence on ppGpp during late stationary phase. PLoS ONE 9:e92690. 10.1371/journal.pone.0092690 24664308 PMC3963941

[B78] Ramachandran V. K. Shearer N. Jacob J. J. Sharma C. M. Thompson A. (2012). The architecture and ppGpp-dependent expression of the primary transcriptome of *Salmonella* Typhimurium during invasion gene expression. BMC Genomics 13:25. 10.1186/1471-2164-13-25 22251276 PMC3293720

[B79] Rogers E. A. Terekhova D. Zhang H. M. Hovis K. M. Schwartz I. Marconi R. T. (2009). Rrp1, a cyclic-di-GMP-producing response regulator, is an important regulator of *Borrelia burgdorferi* core cellular functions. Mol. Microbiol. 71, 1551–1573. 10.1111/j.1365-2958.2009.06621.x 19210621 PMC2843504

[B80] Samuels D. S. (2011). Gene regulation in *Borrelia burgdorferi*. Annu. Rev. Microbiol. 65, 479–499. 10.1146/annurev.micro.112408.134040 21801026

[B81] Samuels D. S. Radolf J. D. (2009). Who is the BosR around here anyway? Mol. Microbiol. 74, 1295–1299. 10.1111/j.1365-2958.2009.06971.x 19943896 PMC3005592

[B82] Samuels D. S. Samuels L. R. N. (2016). Gene regulation during the enzootic cycle of the Lyme disease spirochete. For. Immunopathol. Dis. Therap. 7, 205–212. 10.1615/ForumImmunDisTher.2017019469 29876141 PMC5985821

[B83] Samuels D. S. Drecktrah D. Hall L. S. (2018). Genetic transformation and complementation, in Borrelia burgdorferi: Methods and Protocols, eds Pal U. Buyuktanir O. (New York, NY: Humana Press), 183–200. 10.1007/978-1-4939-7383-5_15 PMC5806694 29032546

[B84] Savage C. R. Arnold W. K. Gjevre-Nail A. Koestler B. J. Bruger E. L. Barker J. R.. (2015). Intracellular concentrations of *Borrelia burgdorferi* cyclic di-AMP are not changed by altered expression of the CdaA synthase. PLoS ONE 10:e0125440. 10.1371/journal.pone.0125440 25906393 PMC4408052

[B85] Sedlazeck F. J. Rescheneder P. von Haeseler A. (2013). NextGenMap: fast and accurate read mapping in highly polymorphic genomes. Bioinformatics 29, 2790–2791. 10.1093/bioinformatics/btt468 23975764

[B86] Serganov A. Nudler E. (2013). A decade of riboswitches. Cell 152, 17–24. 10.1016/j.cell.2012.12.024 23332744 PMC4215550

[B87] Sergiev P. V. Aleksashin N. A. Chugunova A. A. Polikanov Y. S. Dontsova O. A. (2018). Structural and evolutionary insights into ribosomal RNA methylation. Nat. Chem. Biol. 14, 226–235. 10.1038/nchembio.2569 29443970

[B88] Sherlock M. E. Sudarsan N. Breaker R. R. (2018). Riboswitches for the alarmone ppGpp expand the collection of RNA-based signaling systems. Proc. Natl. Acad. Sci. U.S.A. 115, 6052–6057. 10.1073/pnas.1720406115 29784782 PMC6003355

[B89] Sherwood A. V. Henkin T. M. (2016). Riboswitch-mediated gene regulation: novel RNA architectures dictate gene expression responses. Annu. Rev. Microbiol. 70, 361–374. 10.1146/annurev-micro-091014-104306 27607554

[B90] Shi Y. Dadhwal P. Li X. Liang F. T. (2014). BosR functions as a repressor of the *ospAB* operon in *Borrelia burgdorferi*. PLoS ONE 9:e109307. 10.1371/journal.pone.0109307 25271631 PMC4182837

[B91] Steere A. C. Grodzicki R. L. Kornblatt A. N. Craft J. E. Barbour A. G. Burgdorfer W.. (1983). The spirochetal etiology of Lyme disease. N. Engl. J. Med. 308, 733–740. 10.1056/NEJM198303313081301 6828118

[B92] Stevenson B. Seshu J. (2017). Regulation of gene and protein expression in the Lyme disease spirochete, in Current Topics in Microbiology and Immunology, ed B. Adler (Berlin; Heidelberg: Springer). 10.1007/82_2017_49 29064060

[B93] Storz G. Vogel J. Wassarman K. M. (2011). Regulation by small RNAs in bacteria: expanding frontiers. Mol. Cell 43, 880–891. 10.1016/j.molcel.2011.08.022 21925377 PMC3176440

[B94] Sultan S. Z. Pitzer J. E. Miller M. R. Motaleb M. A. (2010). Analysis of a *Borrelia burgdorferi* phosphodiesterase demonstrates a role for cyclic-di-guanosine monophosphate in motility and virulence. Mol. Microbiol. 77, 128–142. 10.1111/j.1365-2958.2010.07191.x 20444101 PMC2907449

[B95] Sze C. W. Smith A. Choi Y. H. Yang X. Pal U. Yu A.. (2013). Study of the response regulator Rrp1 reveals its regulatory role in chitobiose utilization and virulence of *Borrelia burgdorferi*. Infect. Immun. 81, 1775–1787. 10.1128/IAI.00050-13 23478317 PMC3647990

[B96] Thomason M. K. Storz G. (2010). Bacterial antisense RNAs: how many are there, and what are they doing? Annu. Rev. Genet. 44, 167–188. 10.1146/annurev-genet-102209-163523 20707673 PMC3030471

[B97] Trotochaud A. E. Wassarman K. M. (2005). A highly conserved 6S RNA structure is required for regulation of transcription. Nat. Struct. Mol. Biol. 12, 313–319. 10.1038/nsmb917 15793584

[B98] Troxell B. Yang X. F. (2013). Metal-dependent gene regulation in the causative agent of Lyme disease. Front. Cell. Infect. Microbiol. 3:79. 10.3389/fcimb.2013.00079 24298449 PMC3828560

[B99] Vercruysse M. Fauvart M. Jans A. Beullens S. Braeken K. Cloots L.. (2011). Stress response regulators identified through genome-wide transcriptome analysis of the (p)ppGpp-dependent response in *Rhizobium etli*. Genome Biol. 12:R17. 10.1186/gb-2011-12-2-r17 21324192 PMC3188799

[B100] Wang P. Dadhwal P. Cheng Z. Zianni M. R. Rikihisa Y. Liang F. T.. (2013). *Borrelia burgdorferi* oxidative stress regulator BosR directly represses lipoproteins primarily expressed in the tick during mammalian infection. Mol. Microbiol. 89, 1140–1153. 10.1111/mmi.12337 23869590 PMC3772987

[B101] Wassarman K. M. Storz G. (2000). 6S RNA regulates *E. coli* RNA polymerase activity. Cell 101, 613–623. 10.1016/S0092-8674(00)80873-9 10892648

[B102] Waters L. S. Storz G. (2009). Regulatory RNAs in bacteria. Cell 136, 615–628. 10.1016/j.cell.2009.01.043 19239884 PMC3132550

[B103] Yang X. F. Alani S. M. Norgard M. V. (2003). The response regulator Rrp2 is essential for the expression of major membrane lipoproteins in *Borrelia burgdorferi*. Proc. Natl. Acad. Sci. U.S.A. 100, 11001–11006. 10.1073/pnas.1834315100 12949258 PMC196916

[B104] Ye M. Zhang J. J. Fang X. Lawlis G. B. Troxell B. Zhou Y.. (2014). DhhP, a cyclic di-AMP phosphodiesterase of *Borrelia burgdorferi*, is essential for cell growth and virulence. Infect. Immun. 82, 1840–1849. 10.1128/IAI.00030-14 24566626 PMC3993442

