# Supplementary figures and images for: The Stringent Response-Regulated sRNA Transcriptome of Borrelia burgdorferi
Source: Front Cell Infect Microbiol. 2018 Jul 5;8:231. doi: 10.3389/fcimb.2018.00231 (PMC6041397; doi:10.3389/fcimb.2018.00231)

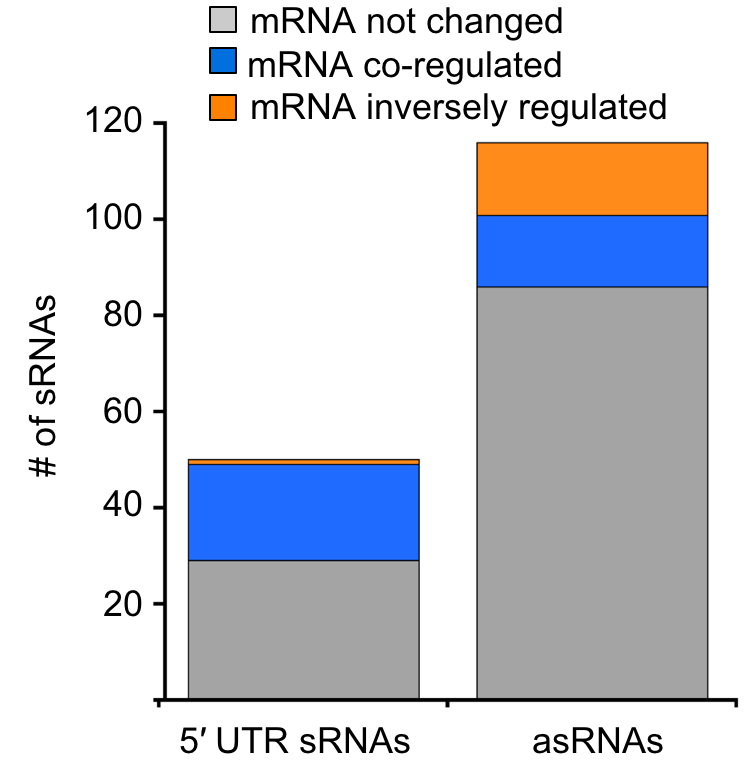

Supplement: Figure S1 — Correlation of RelBbu-dependent sRNAs and cognate mRNAs. The mRNAs associated with RelBbu-dependent 5′ UTR sRNAs or asRNAs were classified as co-regulated (blue bar), inversely regulated (orange bar), or not changed (gray bars) in relation to the sRNA. The correlation was performed using the RelBbu-regulated mRNA transcriptome from Drecktrah et al. (2015). [file Image_1.TIFF]

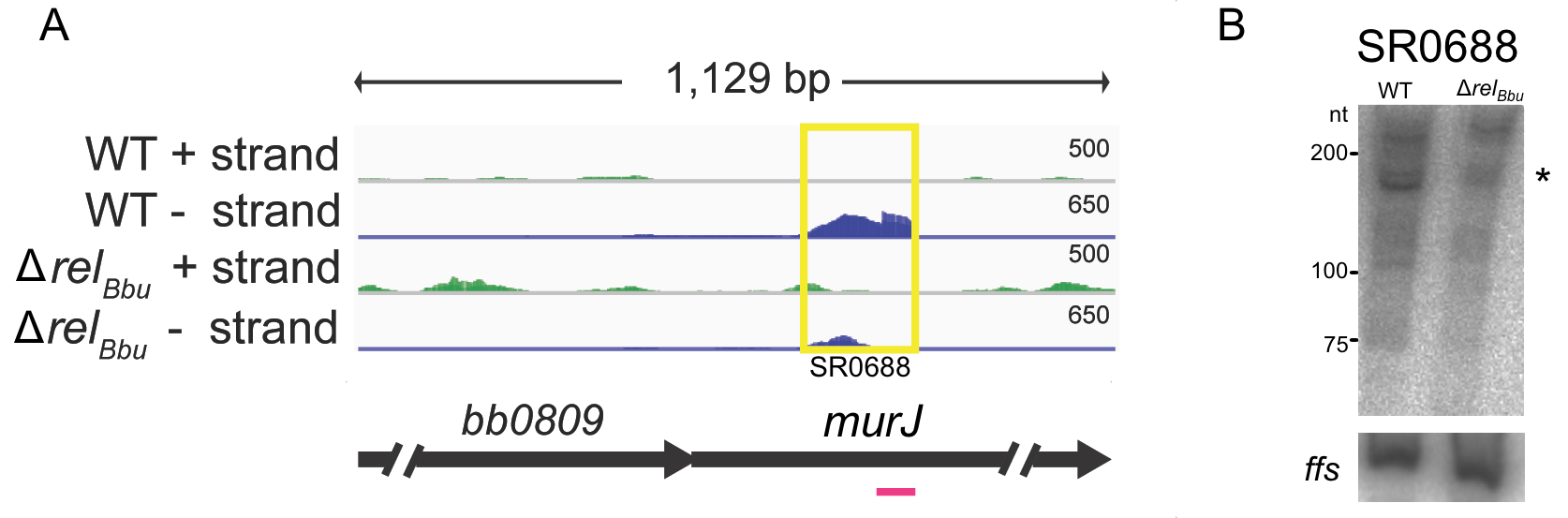

Supplement: Figure S2 — The murJ asRNA (SR0688) is RelBbu-dependent. (A) The deep sequencing reads from both the wild-type and ΔrelBbu strains during nutrient stress are shown in coverage maps with both biological replicates overlaid in dark and light green (plus strand) and dark and light blue (negative strand). The number associated with each strand represents the normalized number of reads mapped and varies depending on the strand. The corresponding ORFs is shown in black below the RNA-seq mapped reads. The yellow box defines the position of the called sRNA with the SR number and the magenta line represents the position of the 32P-labeled oligonucleotide probe used in the Northern blot analyses. (B) Northern blot analyses of total RNA from wild-type and ΔrelBbu strains using a 32P-labeled oligonucleotide probe (Table S1) to the asRNA SR0688 and ffs as a loading control. Asterisk denotes the sRNA size predicted from RNA-seq analyses. [file Image_2.TIFF]

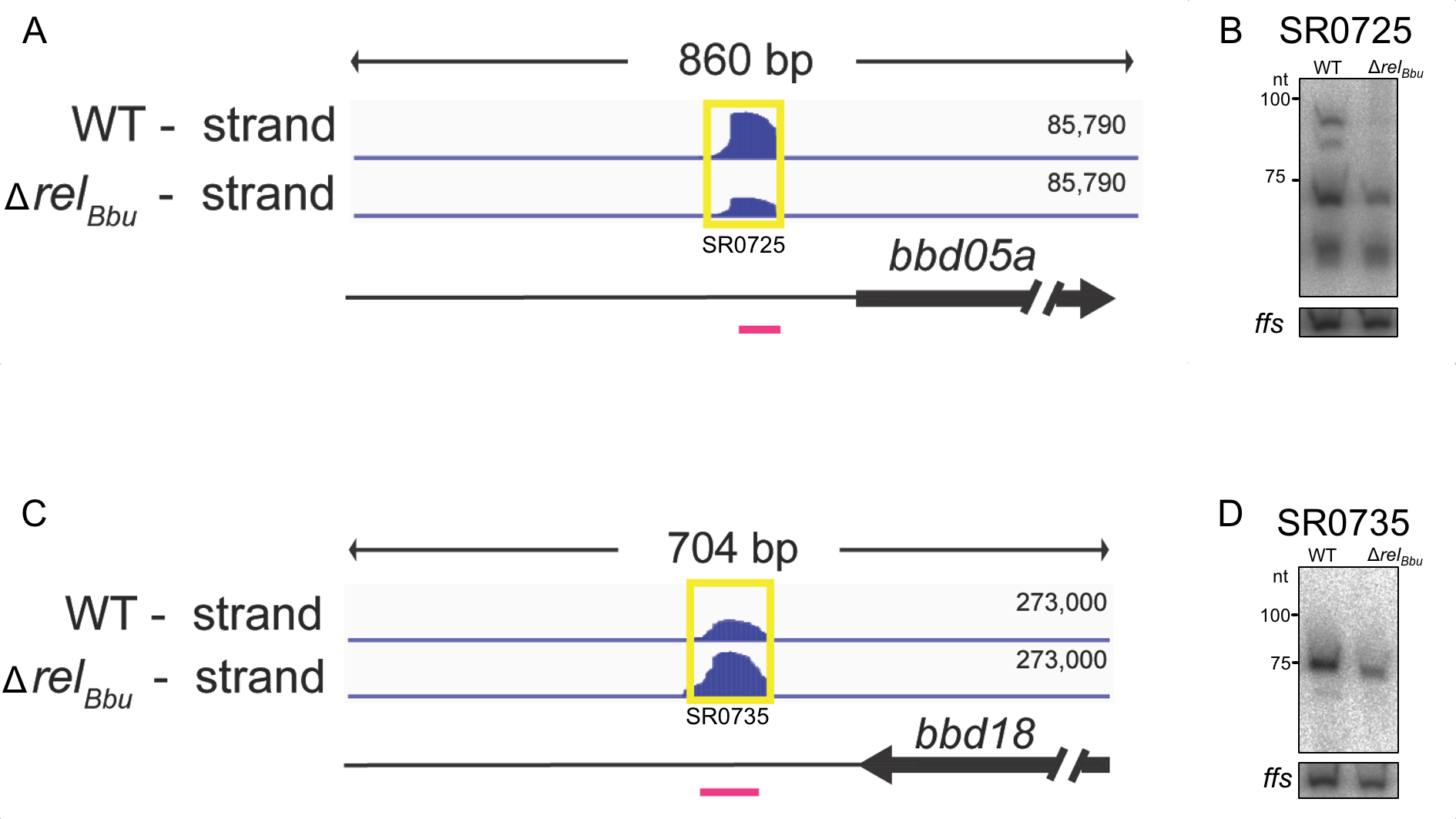

Supplement: Figure S3 — The IG sRNAs SR0725 and SR0735 are RelBbu-dependent. (A,C) The deep sequencing reads from both the wild-type and ΔrelBbu strains during nutrient stress are shown in coverage maps with both biological replicates overlaid in dark and light blue (negative strand). The number associated with each strand represents the normalized number of reads. The corresponding ORFs is shown in black below the RNA-seq coverage maps. The yellow box defines the position of the called sRNA with the SR number and the magenta line represents the position of the 32P-labeled oligonucleotide probe used in the Northern blot analyses. (B,D) Northern blot analyses of total RNA from wild-type and ΔrelBbu strains using a 32P-labeled oligonucleotide probe (Table S1) to the asRNAs SR0725 (B) and SR0735 (D) and ffs as a loading control. [file Image_3.TIFF]

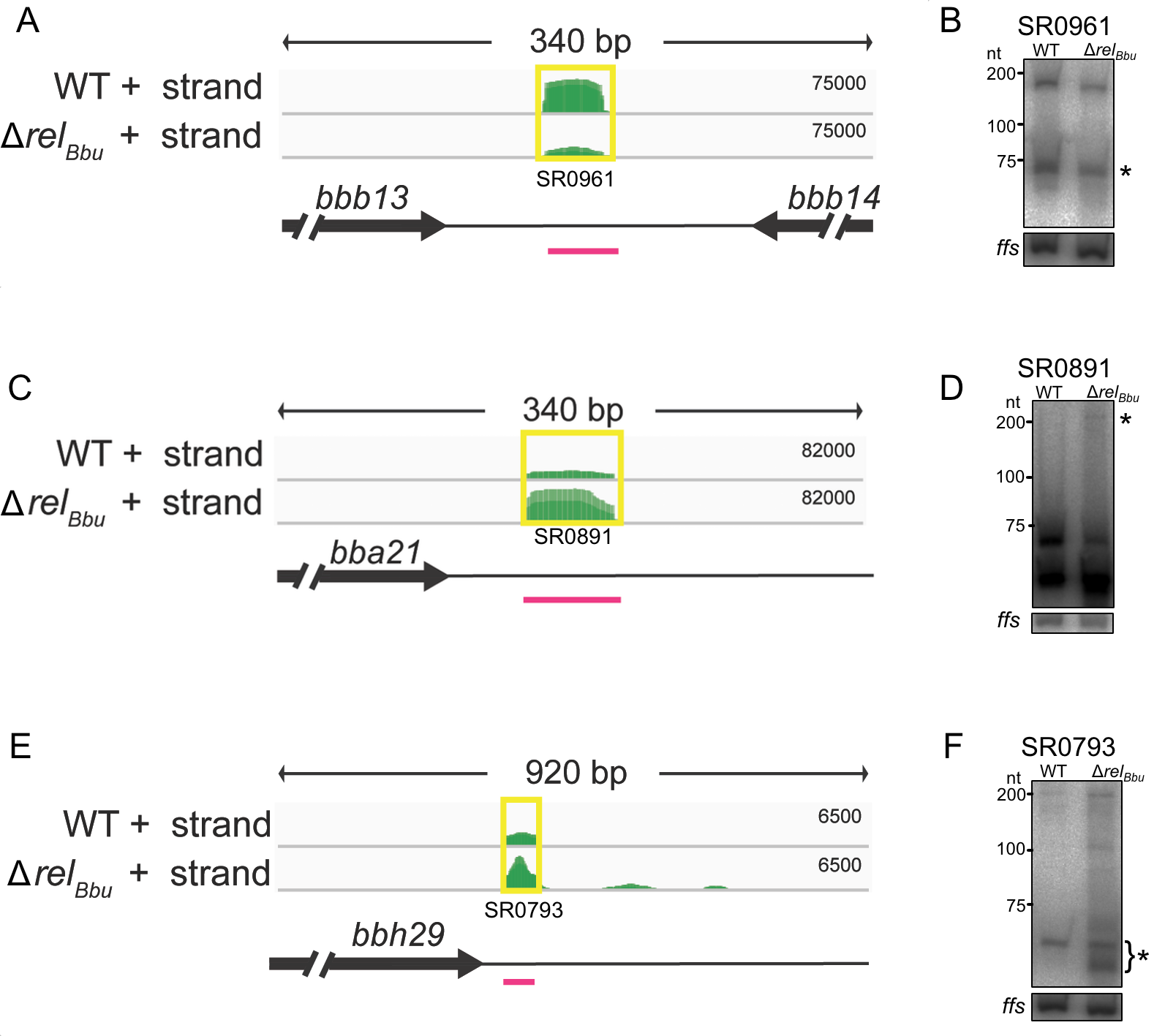

Supplement: Figure S4 — The IG sRNAs SR0961, SR0891, and SR0793 are RelBbu-dependent. (A,C,E) The deep sequencing reads from both the wild-type and ΔrelBbu strains during nutrient stress are shown in coverage maps with both biological replicates overlaid in dark and light green (plus strand). The number associated with each strand represents the normalized number of reads. The corresponding ORFs are shown in black below the RNA-seq coverage maps. The yellow box defines the position of the called sRNA with the SR number and the magenta line represents the position of the 32P-labeled oligonucleotide probes used in the Northern blot analyses. (B,D,F) Northern blot analyses of total RNA from wild-type and ΔrelBbu strains using a 32P-labeled oligonucleotide probe (Table S1) to the asRNAs SR0961 (B), SR0891 (D), and SR0793 (F) and ffs as a loading control. Asterisks denote the sRNA sizes predicted from RNA-seq analyses. [file Image_4.TIFF]
